# Analysis of cell-specific transcriptional responses in human colon tissue using CIBERSORTx

**DOI:** 10.1038/s41598-023-45582-6

**Published:** 2023-10-25

**Authors:** Yueqin He, Julia Nicole DeBenedictis, Florian Caiment, Simone G. J. van Breda, Theo M. C. M. de Kok

**Affiliations:** Department of Toxicogenomics, GROW - School for Oncology and Reproduction, Maastricht University, Universiteitssingel 40, 6229 ER Maastricht, P.O. Box 616, 6200 MD Maastricht, The Netherlands

**Keywords:** Cell biology, Genetics, Gastroenterology, Risk factors

## Abstract

Diet is an important determinant of overall health, and has been linked to the risk of various cancers. To understand the mechanisms involved, transcriptomic responses from human intervention studies are very informative. However, gene expression analysis of human biopsy material only represents the average profile of a mixture of cell types that can mask more subtle, but relevant cell-specific changes. Here, we use the CIBERSORTx algorithm to generate single-cell gene expression from human multicellular colon tissue. We applied the CIBERSORTx to microarray data from the PHYTOME study, which investigated the effects of different types of meat on transcriptional and biomarker changes relevant to colorectal cancer (CRC) risk. First, we used single-cell mRNA sequencing data from healthy colon tissue to generate a novel signature matrix in CIBERSORTx, then we determined the proportions and gene expression of each separate cell type. After comparison, cell proportion analysis showed a continuous upward trend in the abundance of goblet cells and stem cells, and a continuous downward trend in transit amplifying cells after the addition of phytochemicals in red meat products. The dietary intervention influenced the expression of genes involved in the growth and division of stem cells, the metabolism and detoxification of enterocytes, the translation and glycosylation of goblet cells, and the inflammatory response of innate lymphoid cells. These results show that our approach offers novel insights into the heterogeneous gene expression responses of different cell types in colon tissue during a dietary intervention.

## Introduction

Diet is an important, modifiable determinant of cancer risk, and studying the molecular changes induced by dietary factors can aid in the development of cancer prevention strategies. Human dietary intervention studies which aim to investigate the molecular mechanisms involved in cancer development often perform transcriptomic analysis on blood or target tissues collected from participants. However, analyzing multi-cellular tissues such as breast, colon, or lung presents challenges since these tissues consist of a variety of different cell types each with their own specific functions. The nuances in each cell type’s gene expression changes and their role in a coordinated tissue response are lost when gene expression levels are averaged across an entire sample, as is done in bulk-tissue transcriptomic analysis. Additionally, human dietary and lifestyle intervention studies offer more limitations in gene expression research as the data typically contains more noise and variability than what is seen in well controled in vitro studies. The added complexity of free-living individuals contributes to the difficulty of utilizing this study design in mechanistic research. However, these types of studies are crucial for the validation of mechanistic findings from in vitro studies and the translational understanding of diet and cancer risk.

When a single cell sequencing approach is not feasible, machine learning methods for estimating cell-type abundances and cell-type-specific gene expression profiles from bulk tissue transcriptomics may offer a solution. CIBERSORTx^1–3^ is a widely-used algorithm that allows for deeper retrospective analyses of cell type specific gene expression data without physical cell isolation. This digital cytometry method has been validated and used for different purposes^4^.

In the present study, CIBERSORTx analysis is used on data from the PHYTOME study, a human dietary intervention study performed previously at our department, which compared the effects of different meat interventions on transcriptional changes and biomarkers relevant for colorectal cancer (CRC) risk^5^. The association between CRC risk and processed red meat consumption is due to the formation of N-nitroso compounds (NOCs) in the colon. NOCs have mutagenic and genotoxic properties and are formed through the reaction of nitrite and nitrate with amines and amino acids in the presence of heme iron, which is abundant in red meat. Processed red meat contains higher levels of nitrite as food additive, leading to an increased formation of NOCs in the colon^6^. While these foods’ role in CRC risk is well-documented, the impact of adding phytochemicals to these food products to reduce their carcinogenic effect had not been performed previously in a human study on the gene expression level^7–11^. In the PHYTOME study, colon tissue and fecal water samples were collected two weeks after participants consumed either 300 g traditionally processed red meat products or PHYTOME meat products (processed red meat with added phytochemical extracts). A major objective of this study was to measure the change in the amount of N-nitroso compounds (NOCs) in the participants’ fecal water after each intervention phase. Endogenous NOC formation was found to be significantly reduced after consuming PHYTOME meat compared with standard processed red meat, suggesting a protective effect of the phytochemical compounds against colonic NOC formation. The gene expression analysis performed on participant colon tissue, however, did not result in statistically significant differentially expressed genes (DEGs) in colonic tissue when comparing the red meat and PHYTOME meat intervention groups.

This latter outcome is not in line with previous in vitro and animal model studies, where the exposure of similar phytochemical extracts demonstrated significant expression changes related to anticarcinogenic pathways in CRC models^12–14^. We hypothesize that, due to the aforementioned constraints posed by multicellular bulk tissues, a prominent expression signal was not identifiable in the PHYTOME intervention. In this study we therefore aim to establish if cell deconvolution using CIBERSORTx can improve gene expression data analysis to gain novel insights into the cell-specific expression changes in the colon epithelium following the addition of phytochemicals to standard processed red meats. This approach could contribute to a better understanding of red meat-induced carcinogenic processes among different cell types in the colon, and better explain how phytochemicals may intervene in this process. Furthermore, our study also demonstrates the added value of CIBERSORTx in bulk tissue gene expression analysis.

## Methods

### Data source

Single cell RNA sequencing (scRNA-Seq) data of 3 healthy colon samples of the GSE116222 cohort was downloaded from GEO (Gene Expression Omnibus)^15^. We used bulk microarray data from our PHYTOME project GSE147996 cohort^5^, which were log_2_ normalized gene expression values in colonic tissue biopsies from 54 participants. This dietary study included healthy men and women with a body mass index 18–25, in age of 18–70 years. The study’s design encompassed three different, successive meat interventions. The PHYTOME dataset has four time points: T1 refers to the baseline before the intervention; T2 is taken after the participants consumed the provided processed red meat products for two weeks; T3 refers to the time point after a wash-out period of two weeks during which the participants consumed unprocessed chicken and turkey as white meat control group; Finally, T4 refers to the time point after the two-week intervention with PHYTOME meat products (meat products with addition of commercial phytochemicals) at two levels of nitrite: standard-nitrite (T4_B1, n = 25) and reduced-nitrite (T4_B2, n = 28) levels. PHYTOME meat products consisted of cooked sausages, raw and cooked ham, dry fermented sausages, and dry cured ham. Commercial extracts from Polygonum cuspidatum, Sophora japonica, green tea, white grape, rosemary, oregano, sage, Melissa, and acerola were added in meat mince or curing brines, as natural sources of polyphenols and ascorbic acid. During the intervention period, intake of fruits and vegetables were kept at a low, but acceptable level of 50 g of vegetables and one piece of fruit per day.

### Processing and quality control of the single-cell RNA-seq data

FASTQ files from sequence alignments were extracted from the BAM format files which were downloaded from GEO using the tool bamtofastq (version1.4.1)^16^. Then the reads were aligned to the GRCh38 human reference genome using the Cell Ranger toolkit (version 7.0.0)^17^.

Raw count matrices were filtered to only retain genes expressed in at least 10 cells. Cell-level filtering was performed to remove low-quality data including cells with low feature numbers (less than 250), or with low UMI (unique molecular identifier) numbers (less than 500), or with a low number of genes detected per UMI (log10GenesPerUMI < 0.8), or with a high percentage of mitochondrial features (more than 20%) (https://github.com/yueqinhe/CIBERSORTx_P/edit/main/README.md).

Library size normalization was performed using the Seurat R package^18^. A more advanced normalization method, SCTransform, was used for estimating the variance of the filtered data. Then, the three samples were integrated to remove the batch effect using the function IntegrateData of Seurat (4.2.0) (https://github.com/yueqinhe/CIBERSORTx_P/blob/main/Single_cell_analysis).

### Clustering and annotation

Dimensionality reduction was performed using principal component analysis (R, version4.2.1). ElbowPlot was used to estimate the number of relevant principal components in the data, and cells were clustered in the reduced dimensional space using the Seurat package (resolution = 0.4, which was selected because it can avoid unidentified cell types at annotation part). Cell clusters were visualized using UMAP (Uniform Manifold Approximation and Projection).

Annotation was automatically performed using the ScType pipeline^19^. The cell marker file used is the ScType built-in cell marker of the gastrointestinal tract (Supplementary Information 1) modified according to previous publications^15,20^, adding cell types like T cell with marker gene CCL5 and CREM, BEST4/OTOP2 cell with marker gene CA7, BEST4, OTOP2, MT1H, LYPD8, CA4; Mast cell with marker genes shown in “Immune system” class of the file and gene VIM, CD44, TPSAB1 and TPSB2; ILCs with marker genes HLA-DRA, HLA-DQB1, HLA-DPB1, CD74, CD83; Progenitor cells with marker gene SOX9, CDK6, MUC4, FABP5, PLA2G2A, LCN2; and transient-amplifying (TA) cells with marker gene KI67, PCNA, TOP2A, CCNA2, and MCM5. According to the positive and negative markers genes of all cell types within gastrointestinal tract tissue, the cluster summary enrichment-score (called ScType score) were calculated. A cell type with the highest ScType score is used for assignment to each cluster. We consider a negative ScType score to indicate a low-confidence cell-type annotation, which are assigned as “unknown” cell type. The identified cell types were overlayed on a UMAP plot.

### CIBERSORTx

The annotated single-cell RNA-Seq data was uploaded to CIBERSORTx (http://cibersortx.stanford.edu) to create the novel signature matrix with defaulted parameters, except for “Min.Expression” which was set to 0.5 and “Disable quantile normalization” was unchecked. Then, cell fractions were imputed after uploading the normalized and reverse-log transformed values of the bulk tissue microarray data from the PHYTOME study (as explained in a previous paper^5^) with S-mode batch correction and 500 permutations for significance analysis. Finally, cell expression of each cell type was generated using CIBERSORTx’s “Group mode” with “S-mode” batch correction.

### Selection of differentially expressed genes and pathway enrichment

Log_2_FC was generated by “LogFC <—log((geps2$EEC + 1)/(geps1$EEC + 1),2)”, where geps2 refers to gene expression value of T4_B1, and geps1 refers to that of T2. FDR adjusted *p*-values using the Bejamini-Hochberg method, was calculated to correct for multiple comparisons^21^. Then, DEGs were identified using the cut-off thresholds of |log_2_FC|> 1 and FDR < 0.05. Lastly, the DEGs were uploaded to Cytoscape^22^ software 3.9.1 for pathway enrichment analysis using ClueGO^23^ and CluePedia^24^ App with the latest KEGG^25^ pathway database (cut-off threshold of BH adjusted *p* < 0.05). The bubble chart of pathways was visualized by ‘ggplot2’ V3.3.6 package (Wickham, 2016)^22^. For ILCs, gene set enrichment analysis (GSEA)^26^ was performed using KEGG pathways between T4_B1 and T2, and cut-off threshold of BH adjusted P-value was set to 0.05.

### Construction of protein–protein interaction (PPI) network and identification of hub genes

The PPI network of DEGs was constructed in The Search Tool for the Retrieval of Interacting Genes (STRING 11.5; https://string-db.org/), which is an online tool to predict the interactions of genes at the protein level, including direct (physical) and indirect (functional) interactions^27^. Subsequently, the PPI network visualized in Cytoscape and significant modules of the PPI network were selected by the MCODE app with the default parameters (degree cut-off, 2; K-Core, 2; max depth, 100; node score cut-off, 0.2). Moreover, hub genes were selected in Cytoscape with cytoHubba (ranked by degree)^28^.

## Results

### Generation of signature matrix and identification of changes in cell-type proportion

**Figure 1:**
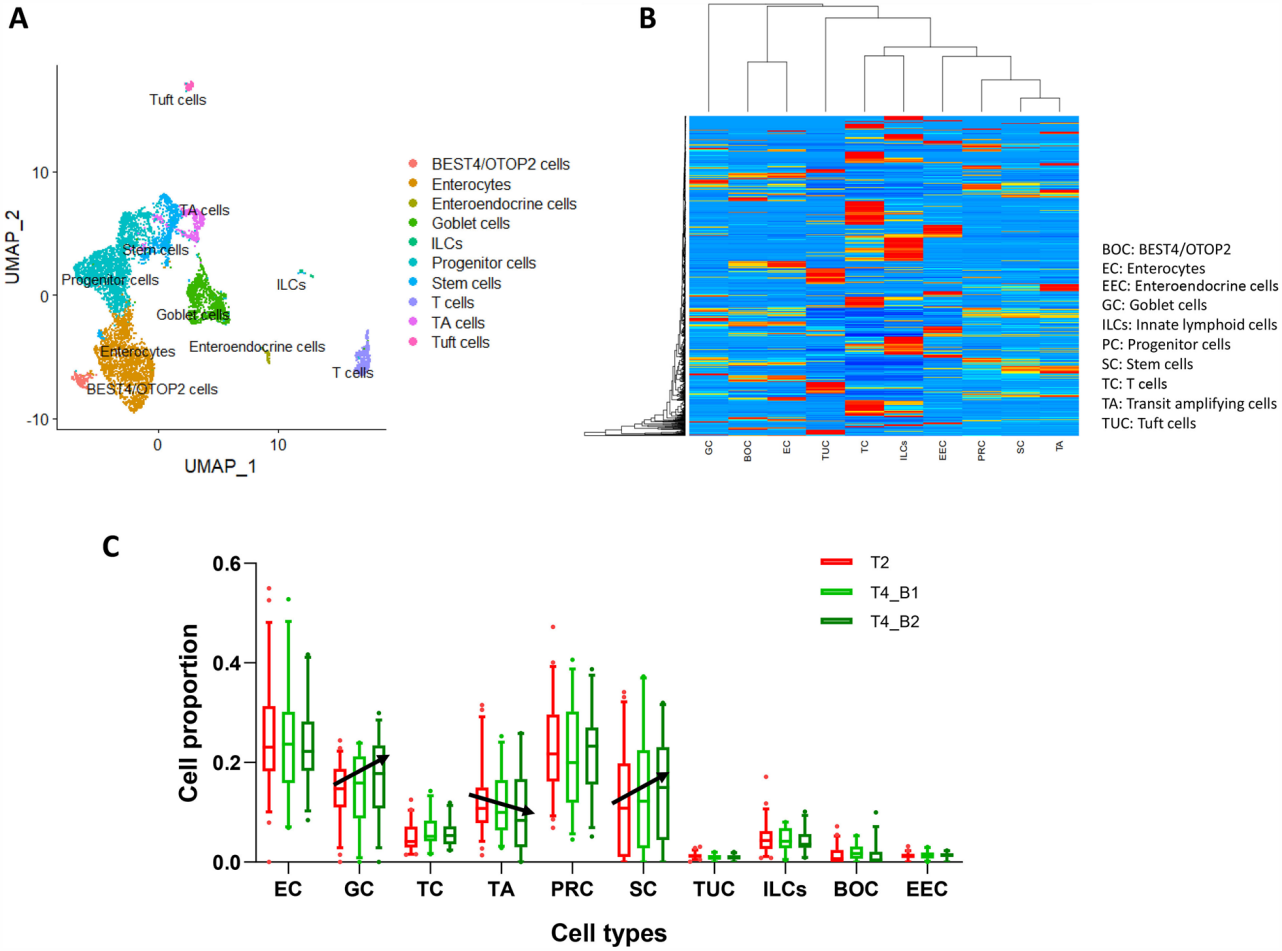
Generation of Novel Signature Matrix and cell proportions. (**A**) Clustering and annotation results of sc-RNASeq data of healthy human colon in UMAP (dim = 17,resolution = 0.4). (**B**) Signature Matrix generated in CIBERSORTx based on annotated sc-RNASeq data. (**C**) Relative cell proportions of bulk colon biopsies collected in T2, T4_B1 and T4_B2 of PHYTOME meat (Ascending arrows indicate continuous upward trends, and a descending arrow indicates a continuous downward trend).

We downloaded raw single-cell RNA-Seq data (10X Genomics, Drop-Seq) from 3 healthy colon samples from GEO^15^. After clustering and annotation, we identified and visualized ten clusters of cells using UMAP (Fig. 1A), which was then uploaded to CIBERSORTx to generate the Novel Signature Matrix. Cell types that share similar biological functions cluster close to each other, as seen in figure (Fig. 1B). The cell-type groups are as follows: undifferentiated cells: stem cells (SC), progenitor cells (PRC) and transit amplifying cells (TA); absorptive cells: BEST4/OTOP2 cells (BOC) and enterocytes (EC); immune cells: innate lymphoid cells (ILCs) and T cells; gel-forming cells: goblet cells (GC); and hormones-secreting cells: enteroendocrine cells (EEC).

To identify the abundance of gene expression changes among each cell type in the human colon after the consumption of processed red meat versus PHYTOME meat, we uploaded the microarray data of T2 and T4 from the PHYTOME study to CIBERSORTx to generate cell proportions based on the aforementioned Signature Matrix. As shown in Fig. 1C, enterocytes represent the highest proportion of the total gene expression as compared to other cell types, followed by goblet cells (when undifferentiated cells are not considered). Furthermore, there is a continuous upward trend in the abundance of goblet cells and stem cells, and a continuous downward trend of TA cells, even though there is no significant difference between the subsequent cell proportions.

### Gene expression changes and involved pathways at single cell-level

In order to investigate the subtle gene expression changes in individual cell types caused by adding phytochemicals to processed red meat, CIBERSORTx’s Group mode was used to generate gene profiles for each cell type, and DEGs of each cell type between T2 and T4_B1 were identified using the cut-off thresholds of |log2FC|> 1 and FDR < 0.05 (Supplementary Information 2). Here, we present the results of the main cell type of each functional category: SC of undifferentiated cell type, and EC of absorptive cell type, GC of gel-forming cell type, ILCs of immune cell type to have an overview of the output of this method.

#### Stem cells: growth and division

**Figure 2:**
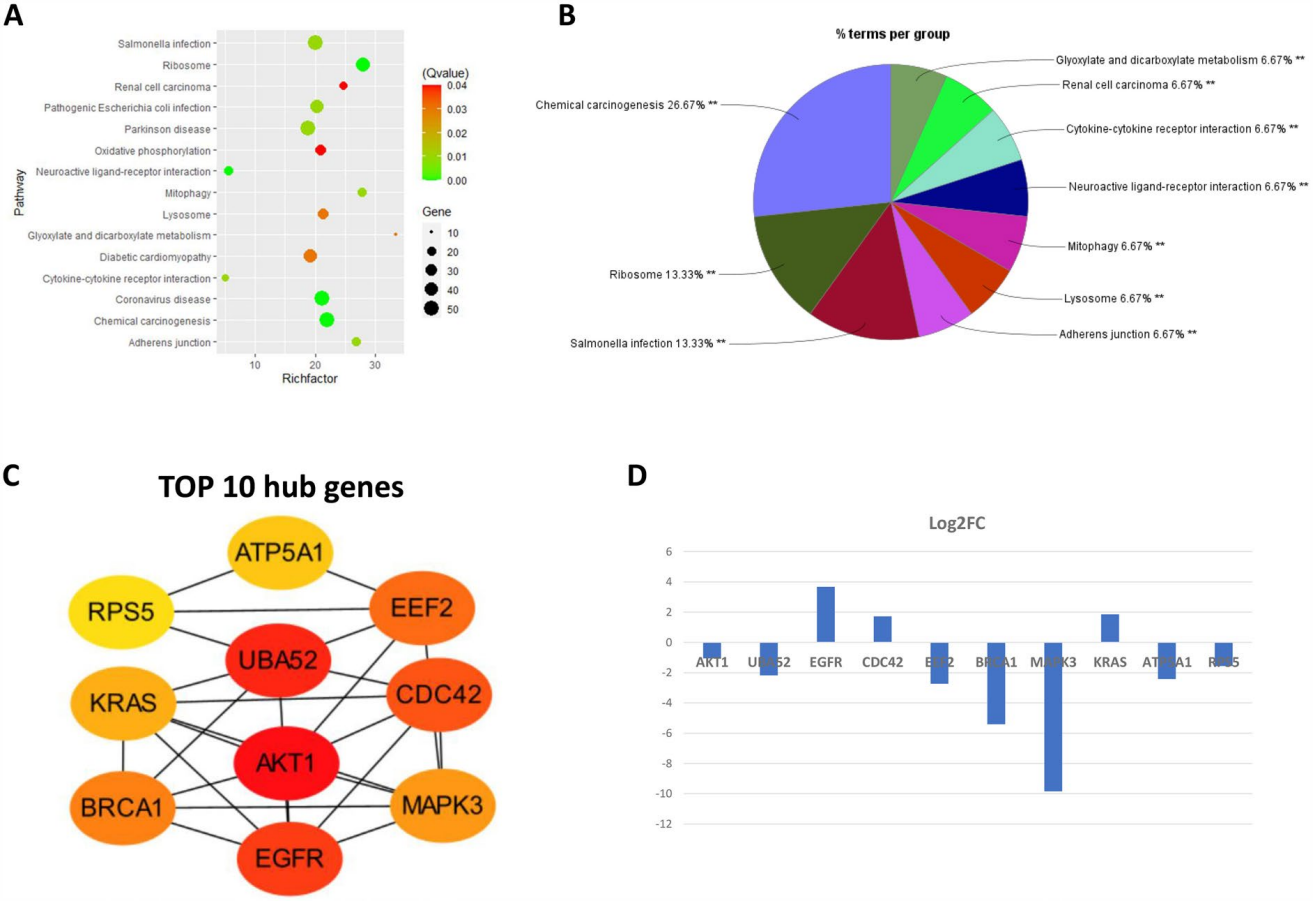
Pathway enrichment and hub gene analysis of DEGs in stem cells. (**A**) Results of KEGG^74^ pathway enrichment (RichFactor refers to the ratio of the number of DEGs present in the KEGG entry to the total number of genes present in that entry. The larger the RichFactor, the greater the degree of enrichment of that pathway). (**B**) Group analysis of pathway terms by Cytoscape (** adjusted group *p* < 0.01). (**C**) Top 10 hub genes generated by cytoHubba ranked with degree (red represent the highest and yellow is the lowest degree of interactions). (**D**) Log2FoldChange of the hub genes.

We found that undifferentiated cell types consistently had a relatively higher number of DEGs as compared with other cell types, especially stem cells (Supplementary Information 3). The DEGs in stem cells of T2 compared with T4_B1 were significantly enriched with KEGG terms related to ribosomes, inflammatory response, adherens junction, and chemical carcinogenesis (Fig. 2A), with the latter being the main grouping term according to the involved DEGs of each group in Cytoscape (Fig. 2B). The top 10 hub genes were identified with STRING and were ranked by degree in Cytoscape (Fig. 2C). Among them, EGFR, CDC42 and KRAS are upregulated in T4_B1 compared with T2, while others are downregulated (Fig. 2D). These genes are known to play important roles in stem cell renewal and will be further described in the discussion.

#### Enterocytes: absorption and metabolism of phytochemicals

**Figure 3:**
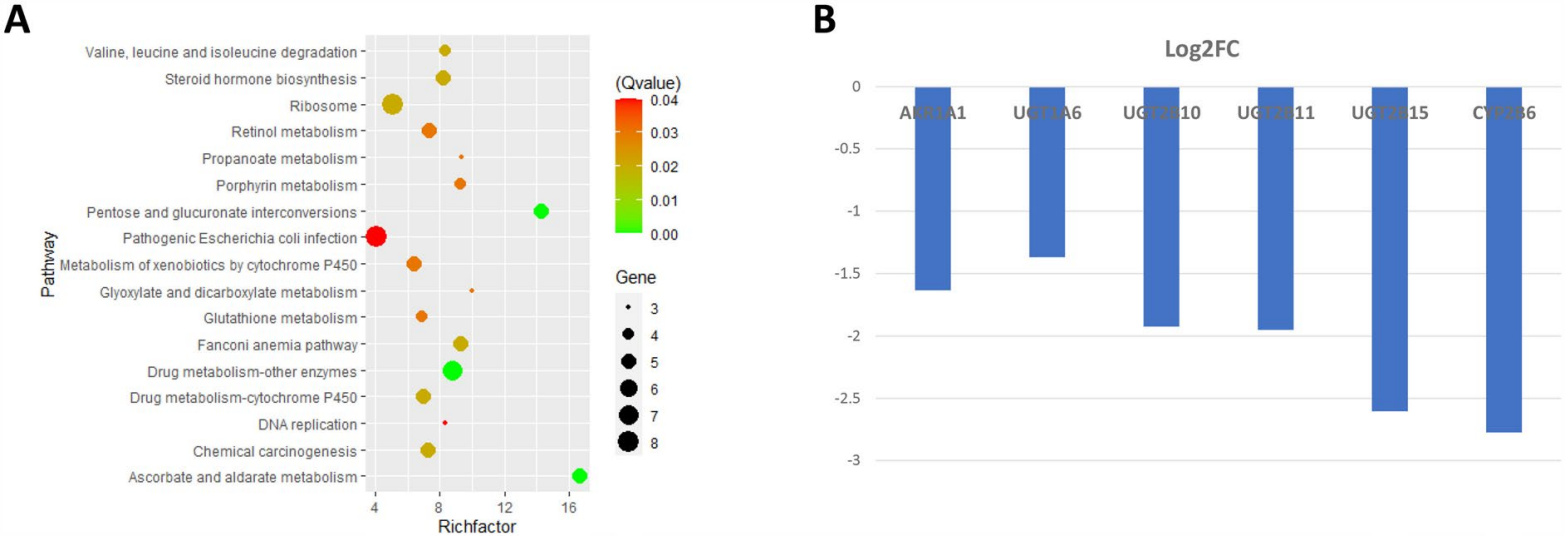
Pathway enrichment and gene changes in enterocyte. (**A**) Results of KEGG pathway enrichment of DEGs in enterocyte when comparing T2 with T4_B1. (**B**) Log_2_ fold change (Log2FC) of the genes involved in the metabolism of vitamin C and A.

The DEGs among the enterocytes were significantly enriched in pathways related to the metabolism of retinol, ascorbate and aldarate. Among them, pathways of pentose and glucuronate interconversions and ascorbate and aldarate metabolism have a relatively high Richfactor (degree of enrichment), above 14, and a Qvalue below 0.01 (Fig. 3A). The expression of the involved genes AKR1A1, UGT1A6, UGT2B10, UGT2B11, UGT2B15 and CYP2B6 are all lower at T4_B1 as compared to T2 (Fig. 3B). Furthermore, drug metabolism and chemical carcinogenesis pathways are also significantly enriched.

#### Goblet cells: translation of mRNA and glycosylation

**Figure 4:**
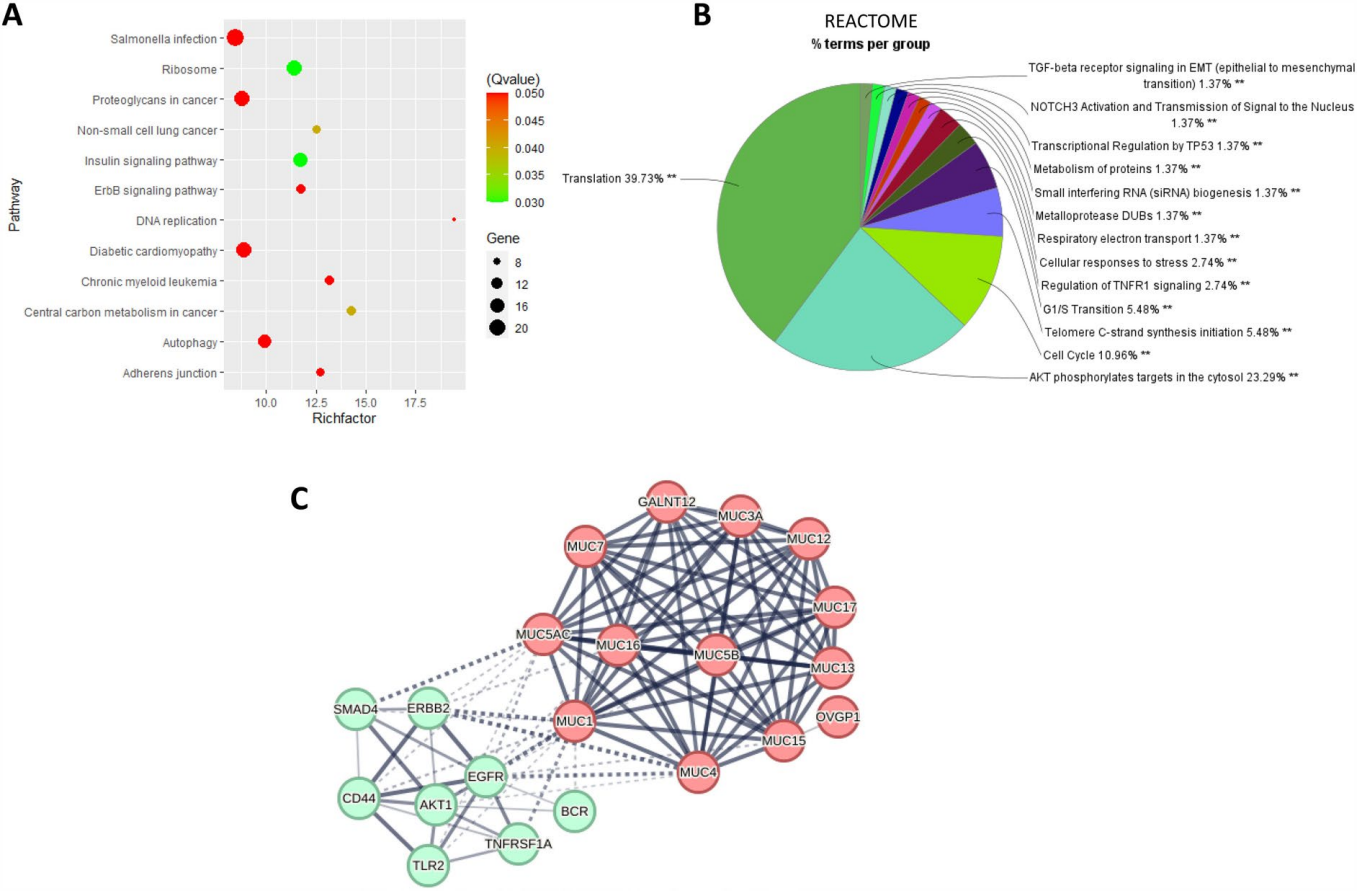
Pathway enrichment and MUC related analysis. (**A**) Results of KEGG pathway enrichment of DEGs in goblet cells when T2 is compared with T4_B1. (**B**) Group analysis of REACTOME enrichment pathway terms by Cytoscape (** adjusted group *p* < 0.01). (**C**) PPI analysis between enriched pathways involved genes (in green) with MUC genes (in red). The solid line represents an inner relation while dotted line is external. Line thickness indicates the strength of data supporting the interaction).

Goblet cells’ DEGs are enriched in pathways like insulin and ribosome signaling, and central carbon metabolism and proteoglycans in cancer, based on the KEGG database (Fig. 4A). Additionally, the REACTOME enrichment results show that mRNA translation pathways are the main cluster, which account for 39.73% of the group’s terms (Fig. 4B). Additionally, immunity-related pathways like the regulation of TNFR1 and infection pathways appear in both results.

To find out which genes in these pathways may contribute to the regulation of the formation of the mucus layer, we analysed the protein–protein interaction (PPI) based on the STRING database. The MUC gene family is highly related to gel forming and membrane-bound^29^. Several MUC genes were selected, then PPI was analysed between the MUC genes and those genes that enriched the pathways. The results in Fig. 4C show the existing connections: EGFR and ERBB2 have strong interactions with MUC1^30,31^ and MUC4^32,33^; MUC5AC with SMAD4; and TNFRSF1A with MUC1. Among the interactions, EGFR phosphorylation has been proven to activate downstream pathways leading to an increase in MUC1 protein expression^31^ and MUC1 is also known to increase the levels and signalling of EGFR in some cellular contexts^11,34,35^.

#### Innate lymphoid cells: inflammatory response

**Figure 5:**
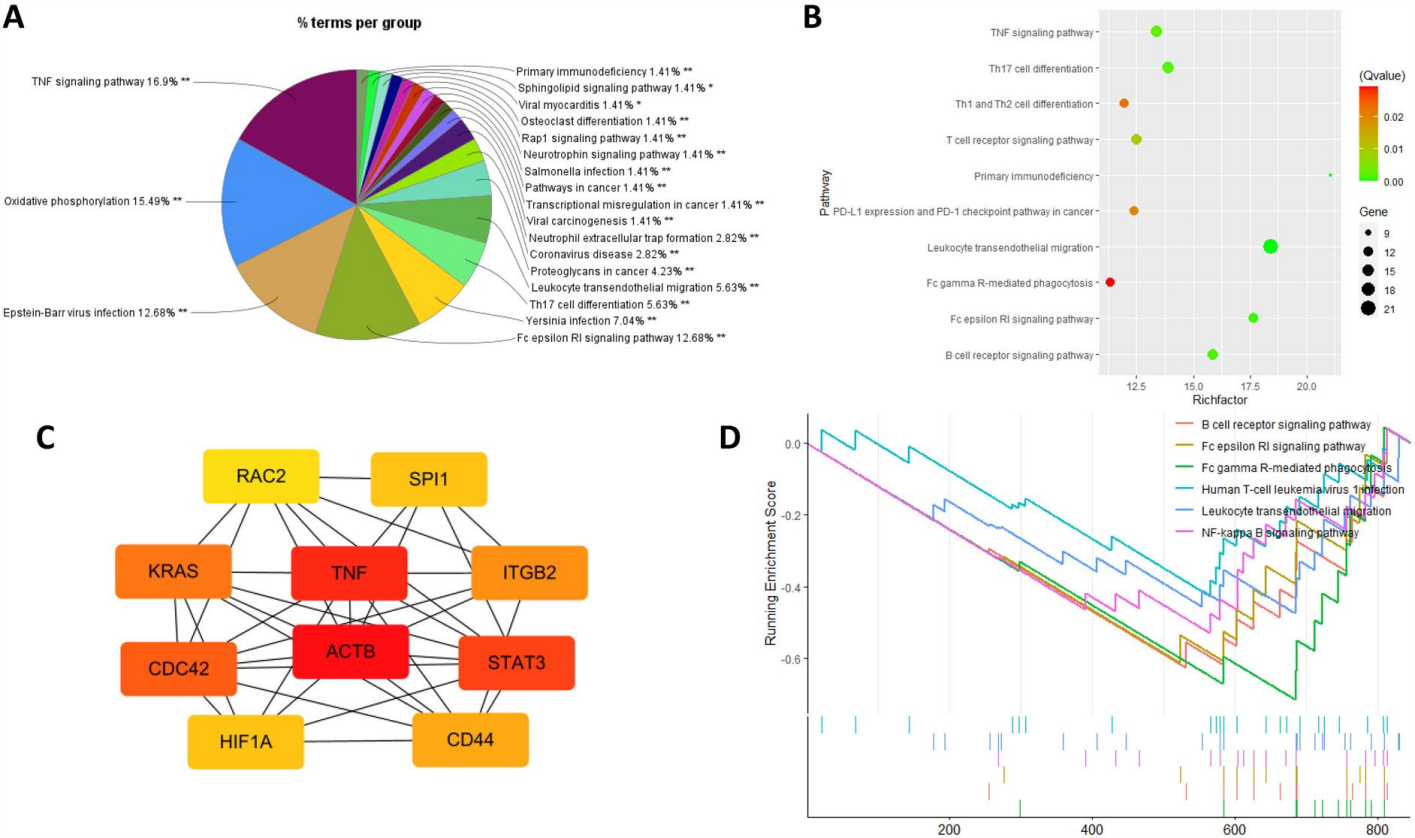
Pathway enrichment and hub gene analysis of DEGs in ILCs. (**A**) Group analysis of KEGG enrichment pathway terms by Cytoscape (** adjusted group *p* < 0.01). (**B**) Immune related KEGG pathway enrichment results of DEGs in ILCs. (**C**) Top 10 hub genes generated by cytoHubba ranked by degree of enrichment (red represents the highest and yellow is the lowest degree). (**D**) Gene set enrichment analysis (GSEA) for immunoreaction pathway.

Innate lymphoid cells (ILCs) play critical roles in regulating intestinal microbe interactions at mucosal barrier surfaces and can be classified into three subgroups, including ILC1, ILC2 and ILC3, with ILC1s similar to Th1 cells, ILC2s similar to Th2 cells and ILC3s similar to Th17 cells^36^. We conducted group analysis of KEGG pathway enrichment results in Cytoscape (Fig. 5A). TNF signaling pathway is the main group term (16.9%), and TNF is also the top one hub gene ranked by degree of enrichment (Fig. 5C). Among the enriched KEGG terms, there are 10 pathways directly related with immune processes (Fig. 5B), like differentiation of Th17, Th1 and Th2, as well as receptor signaling of T cells and B cells. The top 10 hub genes are all downregulated in T4_B1 as compared to T2, as well as the immune related pathways in GSEA analysis (Fig. 5D), like Fc (fragment crystallizable region of antibody) signaling and leukocyte transendothelial migration.

## Discussion

Subtle yet potentially relevant cell-specific gene expression changes may not be identifiable when looking at the results of bulk-tissue gene expression changes alone. Our aim was to extract a more specific picture of the transcriptional changes in colon tissue after a red meat dietary intervention by using bioinformatic techniques and CIBERSORTx deconvolution to identify any changes in cell-specific expression abundance and gene expression. By doing this, we expect to obtain a more precise molecular insight in the mechanistic responses and biological impact on the human colon when adding phytochemicals to processed red meat products.

The changes in cell-specific gene expression abundance showed a continuous upward trend in the proportion of goblet cells (GC) when comparing the processed red meat intervention (T2) with the same meat plus added phytochemicals (T4_B1), and then with a further reduction in nitrite (T4_B2). GCs produce the mucus layer which protects and maintains the integrity of the intestinal lining^37^. Mucus secretion is adapted to respond quickly to insults or infectious agents, and the thinning or dysregulation of the mucus layer is thought to be an early event in the development of chronic inflammatory diseases and intestinal cancer^37,38^. Exposure to carcinogenic agents can damage the intestinal epithelium and disrupt GC function, which can result in reduced mucus secretion and GC depletion^39–42^. However, once the insult is removed, GCs can regenerate quickly and increase mucus secretion to restore the protective barrier of the intestine^43–46^. These results suggests that after a two-week exposure to processed red meat, the addition of phytochemicals to the meat and the reduction of nitrite increasingly stimulated the increasing of GC abundance, potentially in an attempt to recover the protective mucus layer after the increased exposure to N-nitroso compounds, catalytic heme, and cytotoxic aldehydes from the dietary intervention. Moreover, several phytochemicals have been shown to increase GC activity in the intestine. For example, epigallocatechin gallate^47^, resveratrol^48^, quercetin and its derivatives^49–51^, curcumin^52^, and isoflavones^53^, which are included in the commercial extracts used in the intervention^5^, have been shown to increase GC number and/or stimulate production of mucin. Intestinal stem cells (ISC) also play an important role in the regeneration and repair of the intestinal epithelium and taste receptor cells (TA) can modulate the activity of GCs^54–56^. The observed rise in ISC activity may also be related to an increase in ISC differentiation into GCs in order to recover GC loss from the dietary intervention and fortify the mucus layer. Therefore, we speculate that the continuous upward trend in ISC and downward trend in TA expression proportion may be physiologically related to the change in GCs.

Next, the imputation of cell type-specific expression profiles and the analysis of the resulting differentially expressed genes (DEGs) revealed that adding phytochemicals to a diet of processed red meat affected multiple pathways within different cell types, which are consistent with their respective functions and related to CRC risk. Among the 10 cell types, ISCs had the highest number of DEGs and their enriched pathways mostly related to carcinogenesis. Moreover, of the downregulated genes, AKT1 plays a key role in intestinal homeostasis by regulating ISC proliferation, differentiation, and maintenance of function and its activation is a common feature of CRC. Inhibition of AKT1 signaling has been shown to reduce the growth and survival of CRC cells in vitro and in vivo^57^. UBA52, RPS5, and EEF2 are downregulated translation-related genes which respectively code for proteins that regulate the ribosomal protein complex, decode mRNA during translation and ensure accurate ribosome positioning on the mRNA, and assist in ribosomal protein synthesis. Ribosomal proteins have been found to have non-ribosomal functions, and recent studies suggest that these genes may also play a role in stem cell differentiation^58^. Of the upregulated genes, EGFR signaling is well known for promoting ISC growth and division^59^; CDC42 has been reported to play an important role in maintaining cell division of ISCs^60^, and has also been identified as a pharmacological target for ameliorating ISC aging^61^; and KRAS has been found to play a role in regulating intestinal stem cell proliferation and differentiation, as well as the development of CRC^62^. These findings indicate that adding phytochemicals to processed red meat may stimulate the activity of ISCs in the colon, affecting growth, division, and renewal. We speculate that the DEGs and enriched pathways from the ISCs point to an effort of the ISCs to repair the epithelial and mucosal lining after the two-week processed red meat intervention.

The enterocyte (EC) is the most abundant epithelial cell lineage in the human colon, and the apical membrane is characterized by the presence of microvilli^63^. The microvilli form the critical digestive and absorptive interface, a functional microenvironment where enzymes involved in further food breakdown are located and where absorption and transport will occur^64^. After analysis of the DEGs from the aforementioned comparison, the enriched pathways were related to the metabolism of retinol, ascorbate and aldarate, which are consistent with the plant extracts (containing the vitamins A, C and carotenoids) added to the processed red meat intervention. Downregulated genes which were involved in these pathways, like the UGT enzyme family genes, play a critical role in the metabolism and elimination of endogenous and exogenous compounds, including carcinogens^65^. Among which, the UGT1A6 gene expression and activity may contribute to CRC development^66^. In fact, UGT1A6 is involved in the detoxification of heterocyclic amines, pro-carcinogens commonly found in high-heat processed meats^67^. The CYP2B6 gene codes for the cytochrome P450 2B6 enzyme which is part of the body’s first line of defense in detoxifying and breaking down xenobiotics and contribute to a broad range of pro-carcinogen activation reactions^68^. These results suggest that the phytochemicals added to the meat led to an increase in the activity of EC to absorb and metabolize these compounds and a reduction in the detoxification pathways needed during the processed red meat intervention. The reduced expression of UGT1A6 after phytochemical addition may also signal a reduction in CRC risk.

Apart from producing and secreting the intestinal mucus lining, GCs have multiple contributions to innate and adaptive immune responses at mucosal surfaces^69–71^. There are no DEGs related to the formation of mucins like MUC family or CLCA1, FCGBP, AGR2, ZG16, and TFF3, but some of the DEGs like EGFR, SMAD4 and TNFRSF1A all have protein–protein interactions with MUC members, with the thickness of the link lines in the figure representing the number of interactions present between these proteins. Additionally, the proteoglycans pathway was enriched, and the primary constituents of mucins are *O*-glycans which protect the mucus layer against bacterial infection and are important for its high water-binding capacity and gel-forming properties^72^. These results indicate that the phytochemicals may influence mRNA translation and glycosylation for this very important mucus layer-formation.

There are also immunity pathways enriched in the DEGs of GCs, like the regulation of tumor necrosis factor receptor 1 (TNFRSF1A) and infection response. Echoing this, the immune-related pathways enriched by innate lymphoid cells (ILCs), as well as the top ten hub genes, are all downregulated. These genes are normally upregulated with intestinal disorders and contribute to immune cell infiltration and activation of adaptive immunity^73^. These results suggest that adding phytochemicals to processed red meat may decrease pro-inflammatory activities and immune cell activation in the colon.

Taken together, this study provides more information to help us better understand how phytochemicals added to processed red meat influenced gene expression changes in different colon cells. These findings provide a biological basis and potential mechanistic explanations for the reduction in phenotypic markers of CRC risk observed in the original study. Here, our analysis uncovered pathways associated with ISC maintenance, differentiation, and renewal; EC metabolism and detoxification; GC mucus-layer formation; and immune cell function within GCs and ILCs. Except for limited literature interpretation, future studies should focus on validating these findings by utilizing single cell sequencing with a similar dietary intervention design, due to the uniqueness of PHYTOME project and unavailability of the experimental samples. Overall, this approach provided a more detailed and nuanced examination of the gene expression changes in a complex tissue after a dietary intervention that was not possible with bulk RNA transcription analysis alone.

### Supplementary Information

Supplementary Information 1.

Supplementary Information 2.

Supplementary Information 3.

## Data Availability

The microarray data of PHYTOME project have been deposited in the National Center for Biotechnology Information Gene Expression Omnibus under accession number GSE147996. The single cell RNA-sequencing on intestinal epithelial cells from healthy colon deposited in GSE116222.
