# Author Correction: Analysis of cell-specific transcriptional responses in human colon tissue using CIBERSORTx

**DOI:** 10.1038/s41598-024-53927-y

**Published:** 2024-03-01

**Authors:** Yueqin He, Julia Nicole DeBenedictis, Florian Caiment, Simone G. J. van Breda, Theo M. C. M. de Kok

**Affiliations:** Department of Toxicogenomics, GROW ‑ School for Oncology and Reproduction, Maastricht University, Universiteitssingel 40, 6229 ER Maastricht, P.O. Box 616, 6200 MD Maastricht, The Netherlands

Correction to: *Scientific Reports* 10.1038/s41598-023-45582-6, published online 25 October 2023

The original version of this Article contained an error in the spelling of the author Julia Nicole DeBenedictis which was incorrectly given as Julia Nicole De Benedictis.

The original Article has been corrected.

